# Multi-peptide characterization of plasma neurofilament light chain in preclinical and mild Alzheimer’s disease

**DOI:** 10.1093/braincomms/fcae247

**Published:** 2024-08-20

**Authors:** John B Coulton, Yingxin He, Nicolas R Barthélemy, Hong Jiang, David M Holtzman, Randall J Bateman

**Affiliations:** Department of Neurology, Washington University School of Medicine, St. Louis, MO 63110, USA; Tracy Family SILQ Center, Washington University School of Medicine, St. Louis, MO 63110, USA; Department of Neurology, Washington University School of Medicine, St. Louis, MO 63110, USA; Tracy Family SILQ Center, Washington University School of Medicine, St. Louis, MO 63110, USA; Department of Neurology, Washington University School of Medicine, St. Louis, MO 63110, USA; Tracy Family SILQ Center, Washington University School of Medicine, St. Louis, MO 63110, USA; Department of Neurology, Washington University School of Medicine, St. Louis, MO 63110, USA; Knight Alzheimer’s Disease Research Center, Washington University School of Medicine, St. Louis, MO 63108, USA; Hope Center for Neurological Disorders, Washington University School of Medicine, St. Louis, MO 63110, USA; Department of Neurology, Washington University School of Medicine, St. Louis, MO 63110, USA; Knight Alzheimer’s Disease Research Center, Washington University School of Medicine, St. Louis, MO 63108, USA; Hope Center for Neurological Disorders, Washington University School of Medicine, St. Louis, MO 63110, USA; Department of Neurology, Washington University School of Medicine, St. Louis, MO 63110, USA; Tracy Family SILQ Center, Washington University School of Medicine, St. Louis, MO 63110, USA; Knight Alzheimer’s Disease Research Center, Washington University School of Medicine, St. Louis, MO 63108, USA; Hope Center for Neurological Disorders, Washington University School of Medicine, St. Louis, MO 63110, USA

**Keywords:** immunoprecipitation, mass spectrometry, neurofilament, biomarkers, neurodegeneration

## Abstract

Although neurofilament light chain is a well-known marker of neuronal damage, its characterization at the proteoform level is underdeveloped. Here, we describe a new method to profile and quantify neurofilament light chain in plasma at the peptide level, using three in-house monoclonal antibodies targeting distinct protein domains and nano–liquid chromatography coupled to high-resolution tandem mass spectrometry. This study profiled and compared plasma neurofilament light chain to CSF in 102 older individuals (73.9 ± 6.3 years old), 37 of which had a clinical dementia rating greater than 0. We observed elevated neurofilament light chain in preclinical Alzheimer’s disease plasma for two measures (NfL101 and NfL324) and CSF for seven measures (NfL92, NfL101, NfL117, NfL137, NfL148, NfL165 and NfL530). We found five plasma peptides (NfL92, NfL101, NfL117, NfL324 and NfL530) significantly associated with age and two (NfL148 and NfL324) with body mass index.

## Teaser

Coulton *et al*. report a new immunoprecipitation–mass spectrometry assay for plasma neurofilament light chain. They found that profiles of neurofilament light chain differ in CSF and plasma and that different peptides in CSF and plasma separate amyloid groups in preclinical and mild Alzheimer’s disease.

## Graphical Abstract

**Graphical Abstract:**
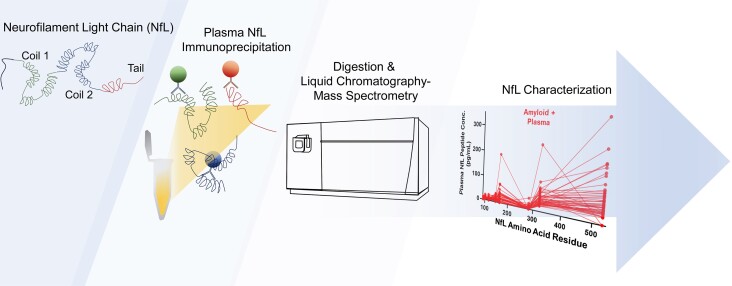

## Introduction

Neurofilament light chain (NfL) in the blood and CSF is a highly useful biomarker of neurodegeneration in multiple diseases.^1,2^ Neurofilament light chain is a structural protein in axons within the central and peripheral nervous systems and is vital to axon calibre and conduction velocity.^3,4^ Neurofilament light chain is released from axons upon neuronal damage; this release increases with healthy aging and rises in CSF and blood in patients from multiple neurodegenerative diseases and is associated with worse clinical outcomes.^5-8^

Neurofilament light chain exists in CSF as a mixture of protein fragments,^9^ but the existence of such complexity is unknown in blood. Mass spectrometry (MS) quantitative mapping of NfL peptides from multiple domains along the NfL protein sequence can characterize proteolytic fragments and assess their utility for quantifying changes related to neurodegeneration. We sought to characterize NfL blood proteoforms changes in preclinical Alzheimer’s disease and assess potential association with corresponding NfL CSF forms.

Early and specific detection of neurodegeneration is necessary for diagnosis, prognosis, monitoring therapeutic treatments and enrolment of participants in research studies, including clinical and prevention trials.^10,11^ A neurodegeneration test to support and monitor therapeutic development requires high specificity and sensitivity for staging disease and differentiate participants with normal and mild cognitive impairment. Such test should be minimally invasive and inexpensive. Currently, NfL is a promising biomarker of neurodegeneration, with a commercially available immunoassay already utilized for monitoring clinical cohorts and trials. While the NfL immunoassay has been successful at distinguishing neurodegenerative diseases from healthy control groups, the biomarker’s utility is currently limited by its non-specific nature and lack of effect size in Alzheimer’s disease.^1^ Increases of NfL due to aging, and multiple central and peripheral neurodegenerative and neuroinflammatory processes, makes NfL a less reliable marker of disease status and treatment response.^2,7,12^ Further, while NfL undergoes multiple post-translational modifications, including proteolytic fragmentation, the immunoassay only measures one epitope of NfL.^13^

Mass spectrometry offers several advantages compared to immunoassays, including broader coverage of all regions of the protein with improved specificity (for NfL proteoforms) and improved precision with ease of multiplexing, measuring multiple analytes at the same time. We previously described NfL proteoforms in brain and CSF^9^ and designed this study to address the effects of amyloidosis in both CSF and blood NfL. Here, we describe our efforts to expand the utility of the previous mass spectrometric assay to characterize and quantify NfL proteoforms in the 40-fold dilute, more complex matrix of blood plasma. We assessed the utility for staging and comparing NfL immunoprecipitation–MS (IP–MS) to other biomarker and clinical measures, including PET measures of amyloid plaques, CSF Aβ42:40, clinical staging with Clinical Dementia Rating–Sum of Boxes (CDR–SB) and tau pathophysiology as measured by tau biomarkers, such as CSF % p-tau217. Further, these findings will enable future physiologic studies on the biology of NfL processing.

## Materials and methods

### Plasma sample handling, preparation, and immunoprecipitation

Archival plasma samples from a previous study of amyloid kinetics were selected based on availability blinded to the other results of the participant such as amyloid and clinical status; sample collection protocols have been described previously.^14^ Briefly, plasma and CSF were collected at baseline and hourly following a bolus infusion of isotopically labelled leucine (^13^C_6_-L). CSF and blood samples were immediately frozen (−80°C) upon collection in 1 mL polypropylene tubes. For sample preparation, EDTA plasma samples (1.0 mL) were thawed at room temperature and centrifuged (21 000 × *g*, 4°C, 30 min). Following centrifugation, plasma aliquots (900 µL) were transferred to microcentrifuge tubes (1.7 mL) and mixed with pre-aliquoted recombinantly labelled NfL internal standard (ISTD; U-^15^N-NfL, Promise Proteomics, Grenoble, France), 0.5 ng per IP). Mixed ISTD plasma aliquots were vortexed, briefly centrifuged and transferred to a 96-well plate array. An aliquot (50 µL) of master mix containing chaotropic agents (5 mM guanidine hydrochloride), protease inhibitor (1×, Roche complete PI cocktail) and surfactant (IGEPAL 1%) was added to each sample, followed by in-house anti-NfL antibody magnetic bead slurry (30 µL per sample, 1:1:1 mix of HJ30.4, HJ30.11 and HJ30.13, targeting Coil 2B, Tail subdomain and Coil 1A, respectively, coupled to Dynabead M-270 Epoxy, Thermo Fisher, Waltham, MA, using manufacturer’s instructions^9^). Samples were IP overnight at 4°C via Thermoshaker (1000 rpm, 16 h, Eppendorf, Leipzig, Germany, equipped with 96-well plate housing) and 2 h at room temperature the following day using a Kingfisher Flex Station sample purification system (Thermo). Briefly, plasma IP samples were mixed by magnetic tip-bead comb array for 2 h at room temperature; then, magnetic beads were isolated from plasma samples and rinsed three times with triethylammonium bicarbonate (TEABC; 25 mM) to remove non-specifically bound species. After washing, post-IP beads were dispensed in an aliquot of buffer for on-bead digestion (25 mM TEABC, 100 µL per well). Bound species on post-IP beads were digested with trypsin/lys-c (400 ng/sample, MS grade, Promega Corp. Madison, WI) overnight at 37°C. Resultant tryptic peptides were cleaned via solid-phase extraction using Sep-Pak C18 µ-Elution plate (Waters Corp.) coupled to a vacuum manifold (Waters Corp.). Briefly, solid phase extraction (SPE) material was wetted with organic solvent (150 µL, 60% ACN, 0.1%FA) and re-equilibrated with aqueous solvent (2 × 150 µL, 0.1%FA) prior to loading resultant peptides. Samples were loaded and washed with aqueous solvent (3 × 150 µL, 0.1%FA) and eluted from C18 with organic solvent (2 × 50 µL, 60%ACN, 0.1%FA). Eluates were transferred to microcentrifuge tubes and solvent was evaporated *in vacuo*.

### CSF sample handling, preparation, and immunoprecipitation

Archival CSF samples from a previous study of amyloid kinetics were selected based on availability blinded to the other results of the participant such as amyloid and clinical status; sample collection and handling protocols are previously described.^14^ CSF aliquots (450 µL) were thawed at room temperature, transferred to microcentrifuge tubes (1.7 mL) and mixed with pre-aliquoted recombinantly labelled NfL internal standard (U-^15^N-NfL, Promise Proteomics, 0.5 ng per IP). Mixed ISTD CSF aliquots were vortexed, briefly centrifuged and transferred to a 96-well plate array. An aliquot (25 µL) of master mix (composition described above) was added to each sample, followed by in-house anti-NfL antibody magnetic bead slurry (30 µL per sample, 1:1:1 mix of HJ30.4, HJ30.11 and HJ30.13, coupled to Dynabead M270-Epoxy, Thermo). Samples were IP for 2 h at room temperature on a Kingfisher Flex Station (Thermo) as described above. Following IP, coupled beads were digested with trypsin/lys-c (400 ng/sample, MS grade, Promega Corp.) at 37°C for 16 h. Resultant tryptic peptides were cleaned via solid-phase extraction using hydrophilic-lipophilic balance (HLB) (Waters Corp.) coupled to a vacuum manifold (Waters). Briefly, SPE material was wetted with organic solvent (150 µL, 60% ACN, 0.1%FA) and re-equilibrated with aqueous solvent (2 × 150 µL, 0.1%FA) prior to loading tryptic digests of NfL. Peptides were loaded and washed with aqueous solvent (3 × 150 µL, 0.1%FA) and eluted from stationary phase with organic solvent (2 × 50 µL, 60%ACN, 0.1%FA). Eluates were transferred to microcentrifuge tubes and solvent was evaporated *in vacuo*.

### Liquid chromatography–mass spectrometry of tryptic neurofilament light chain peptides from CSF and plasma

Resultant samples were reconstituted in 0.1%FA and analysed via nano–liquid chromatography–MS/MS. Tryptic peptides were injected (4.5 µL aliquot) by an M-Class nano-Acquity LC (Waters, Milford, MA) fitted with an HSS C18 T3 analytical column (75 µm × 100 µm, 1.8 µm particle diameter). Samples were loaded on column via direct inject at 0.7 µL/min, with mobile phase composition 99.5% A (0.1% formic acid) and 0.5% B (ACN, 0.1%FA) from *t* = 0 to 7.5 min. Sample mixtures were separated at 0.4 µL/min with the following gradient: *t* = 7.6 min (%A, 99.5; %B, 0.)5; *t* = 7.7 min (%A, 94; %B, 6); *t* = 24 min (%A, 66; %B, 34); *t* = 25 min (%A, 5; %B, 95); *t* = 26.99 min (%A, 5; %B, 95); and *t* = 27 min (%A, 99.5; %B, 0.5). Neurofilament light chain peptides were analysed in positive ion mode, spray voltage was 2.2 kV, and ion transfer tube temperature was 275°C. Parallel reaction monitoring (PRM) was employed for monitoring specific MS/MS transitions of endogenous and isotopically labelled (U-^15^N) NfL peptides.

### Statistical analysis

Mass spectrometry data were extracted using Skyline 22.1,^15,16^ and peak area ratio to ITSD and sample volume were used to derive endogenous NfL concentrations in plasma and CSF. Monitored peptides were confirmed to be NfL-specific by cross-reference of sequences with UniProt database (screened against *Homo sapiens* reviewed UniProt database) and comparison of endogenous peptide fragment ions to corresponding ITSD (U^15^N-labelled, Promise Proteomics) and AQUA peptide (Thermo Fisher Scientific) fragment ions. Mass spectrometry signals within 10 ppm of theoretical *m*/*z* were considered mass accurate. Figures were generated using RStudio 2021.09.1, Integrated Development for R. (RStudio, PBC, Boston, MA) and GraphPad Prism 10.0.2. Cut-off for amyloid status determination by Aβ 42/40 was calculated using amyloid status by Pittsburgh Compound B PET via area under the curve (AUC) analyses performed in both GraphPad Prism and RStudio using package pROC.^17^ Statistical tests comparing NfL levels of amyloid groups (unpaired *t*-test with Welch correction, α = 0.05) were performed using GraphPad Prism. Scatterplots and Pearson correlations were generated in RStudio with ggplot2^18^ and in GraphPad Prism; *P* values denote whether linear slopes are significantly different from zero. Correlograms were generated in RStudio using corrplot package.^19^

### Study design: proof of principle and validation cohort characteristics

Plasma aliquots (*n* = 4 from each participant) from one amyloid-positive participant and one amyloid-negative participant were IP for NfL using a mixture of in-house antibodies targeting three domains across the protein. The researcher performing the experiment was blinded to amyloid status from sample preparation to data analysis. Archival biofluid samples were obtained, prepared and analysed by blinded researchers. Given this is a retrospective study, sample size calculations were not performed to determine required statistical power prior to analysis, and since samples were selected in a blinded fashion to the other results of the participant such as amyloid and clinical status and based only on availability, no exclusion criteria were pre-determined for sampling archival cohorts. Samples studied were from previous studies of amyloid kinetics, with all human studies approved by the Washington University Internal Review Board. In absence of Pittsburgh Compound B PET scores, amyloid status was determined by CSF Aβ 42/40. Cohort demographics are provided in Supplementary Table 1. Average participant age was 73.9 ± 6.3 years (amyloid negative, 73.5 ± 5.9; amyloid positive, 74.3 ± 6.6), contained 40 female participants (amyloid negative, 19; amyloid positive, 21), an average BMI of 27.2 ± 5.3, 37 participants with CDR > 0 and an average CDR–SB of 1.1 ± 2.0 (amyloid negative, 0.5 ± 1.1; amyloid positive, 1.8 ± 2.5). Participants with amyloid-positive CSF and CDR 0 were considered preclinical Alzheimer’s disease (*n* = 25), and participants with amyloid-positive CSF and CDR ≥ 0.5 were considered mild clinical Alzheimer’s disease (*n* = 23 total; CDR 0.5, *n* = 18; CDR 1, *n* = 7). STROBE cross-sectional reporting guidelines were used in this study.^20^

## Results

### Plasma neurofilament light chain by immunoprecipitation–mass spectrometry shows elevated neurofilament light chain levels in samples from participants with amyloid plaques

**Figure 1:**
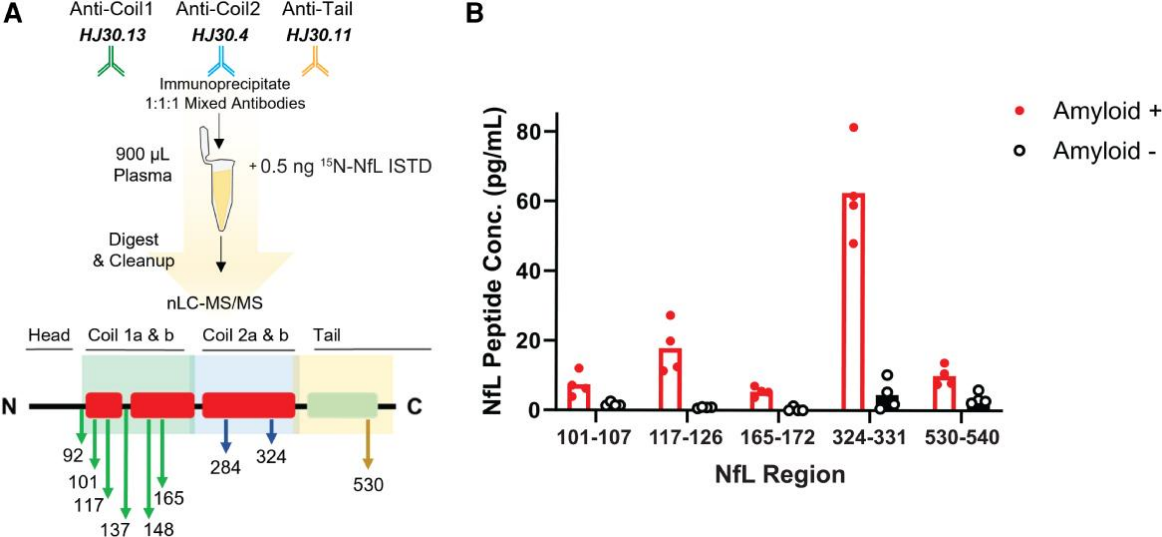
**Proof of principle showing successful quantitation of NfL proteoforms in plasma.** Alzheimer’s disease (*n* = 1 individual, four replicate experiments) and control (*n* = 1 individual, four replicate experiments) plasma NfL were profiled by IP–MS. (**A**) Schematic diagram of IP–MS method for plasma NfL quantitation and profiling. (**B**) Successful IP–MS quantitation of plasma NfL for five regions or proteoforms. Bars represent the mean NfL peptide concentration from four separate IP–MS experiments with dots as individual values.

Pooled plasma samples for amyloid-positive and amyloid-negative controls were obtained to determine the feasibility of NfL measures by IP–MS (Fig. 1). Neurofilament light chain concentrations of the five peptides monitored were increased in amyloid-positive plasma, and we show IP–MS allows for profiling along multiple protein domains (Fig. 1B) of plasma NfL.

### Neurofilament light chain profiles of preclinical Alzheimer’s disease

#### Validation cohort

**Figure 2:**
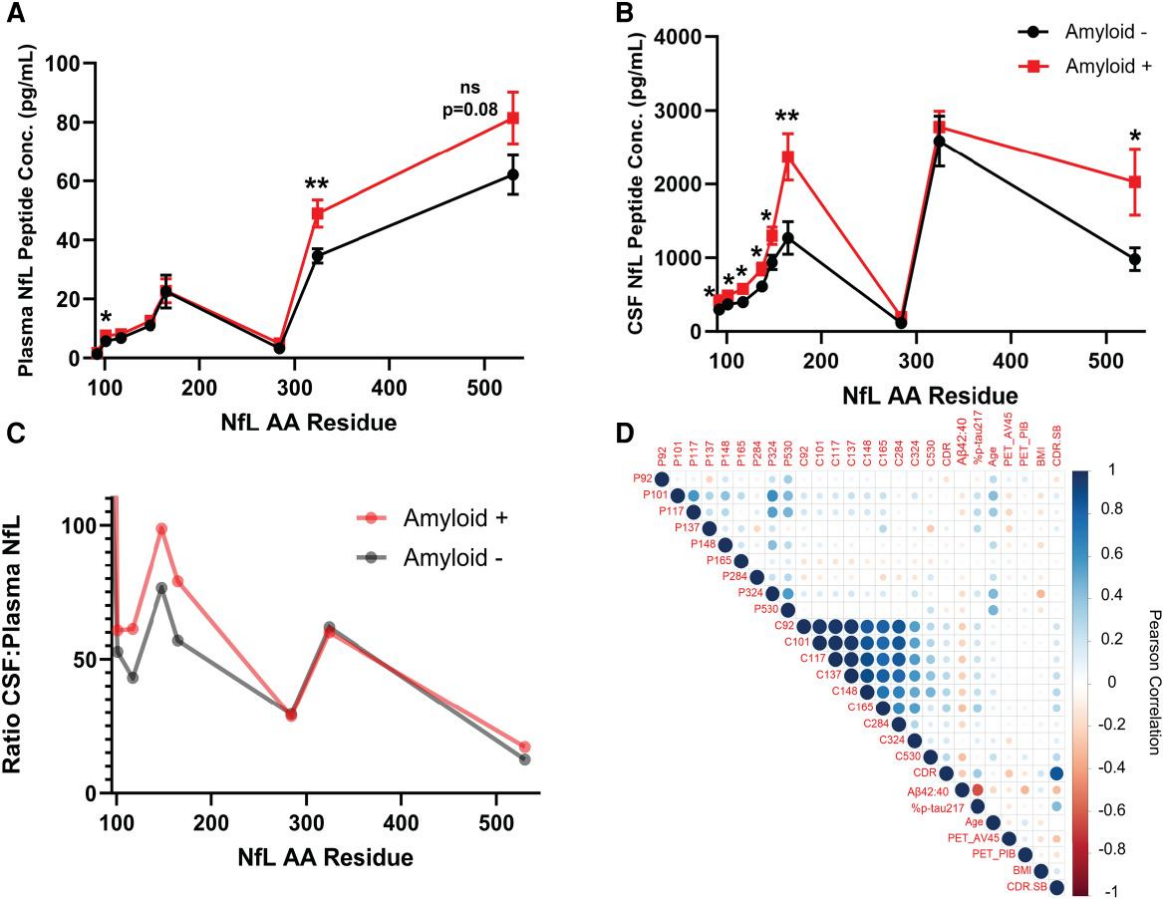
**Plasma NfL peptide profile differs from corresponding CSF NfL profile.** IP–MS profiles of NfL peptide concentrations in plasma and CSF from amyloid-negative and amyloid-positive participants demonstrate different profiles and regional differences between amyloid-positive and amyloid-negative groups indicating that NfL proteoforms are different between CSF and plasma and have different effect sizes in detecting changes due to amyloid plaques. Averaged NfL profiles in **(A)** plasma and **(B)** CSF. **(C)** Median CSF-to-plasma NfL ratios by amyloid group. **(D)** Pearson correlogram of plasma and CSF IP–MS measures with CDR and fluid biomarkers. [**A** and **B** represent mean peptide concentrations and error bars are standard error of the mean (SEM), *n* = 50 amyloid −; *n* = 52 amyloid +]. *P < 0.05 and **P < 0.01, ‘ns’, not statistically significant. Statistical significance determined by an unpaired *t*-test with Welch correction, α = 0.05, between individual peptide-level comparisons (e.g. NfL92 level compared in amyloid-negative and amyloid-positive groups). P, plasma peptides of NfL followed by the N-terminal amino acid residue; C, CSF peptides of NfL followed by the N-terminal AA residue; Aβ42:40, beta 42 to 40 ratio in CSF; %p-tau217, phosphorylation occupancy on threonine 217 of tau in CSF; AV45, denotes florbetapir; PIB, Pittsburgh Compound B; BMI, body mass index; CDR–SB, Clinical Dementia Rating–Sum of Boxes.

Plasma aliquots and matching CSF were selected from an archived, in-house cohort to study late-onset Alzheimer’s disease as previously described.^21,22^ The cohort (additional demographic information in Supplementary Table 1) included 105 participants with an average age of 73.9 (±6 years), with 53 being amyloid positive and 52 being amyloid negative (as determined by CSF Aβ42/40 cut-off of 0.083). Neurofilament light chain peptides (eight in plasma and nine in CSF) were quantified to generate profiles in plasma and CSF samples from amyloid-positive and amyloid-negative participants (Fig. 2). No statistically significant differences were found between means of amyloid-positive and amyloid-negative groups by age, sex, or body mass index (BMI; Supplementary Fig. 1).

#### Multi-peptide plasma neurofilament light chain quantification by immunoprecipitation–mass spectrometry demonstrates neurofilament light chain truncation

Here, we demonstrate that NfL detection and quantification in plasma (0.9 mL, by optimized IP–MS methods previously described by our group)^9^ can be used to generate profiles to compare NfL peptides in diseased and non-diseased populations. The relative peptide amounts observed (Figs. 1 and 2) support plasma NfL truncation, similar to our previous observations in brain tissue (two fragments) and CSF (three main fragments).^9^

#### Neurofilament light chain has different protein profiles in plasma compared to CSF

We find that NfL profiles differ in CSF and plasma in amyloid-negative individuals, as well as those with preclinical and mild Alzheimer’s disease. Our profiling of NfL shows that the peptides that are useful for separating amyloid groups are different for plasma and CSF. In plasma, we see statistically significant separation of amyloid groups in Coil 2B of the rod domain (NfL324; NfL peptide GMNEALEK spanning residues 324–331), which is not significantly different in the CSF (*P* = 0.007 and 0.6, respectively). Conversely, in CSF, we observe the C-terminal subdomain peptide (NfL530; NfL peptide VEGAGEEQAAK spanning residues 530–540) is significantly increased in amyloid-positive participants (*P* = 0.03) but not in plasma (*P* = 0.08). Though these data alone are not sufficient to draw strong conclusions, it may imply that NfL concentrations in plasma or serum are not only a result of simple dilution, or that peripheral contributions of C-terminal NfL in plasma dilute the differences observed in centrally derived NfL530 in CSF.

#### Ratio of CSF neurofilament light chain to plasma neurofilament light chain shows Coil 1 peptides are relatively increased in amyloid-positive participants

We generated simple ratios of CSF-to-plasma NfL concentrations for matched samples to elucidate relative differences between CSF and plasma NfL by amyloid status (Fig. 2C). Median ratios of CSF-to-plasma NfL show that Coil 1 peptides [NfL101 (NfL peptide FASFIER spanning residues 101–10)], NfL117 (NfL peptide VLEAELLVLR spanning residues 117–126), NfL148 (NfL peptide LAAEDATNEK spanning residues 148–157) and NfL165 (NfL peptide EGLEETLR spanning residues 165–172)] are relatively increased in amyloid-positive CSF compared to amyloid-negative CSF.

#### Regional neurofilament light chain profiles are more correlated in CSF compared to plasma

Neurofilament light chain IP–MS measures in CSF and plasma showed mostly weak correlations (Fig. 2D). Coil 1A and 2B peptides in plasma exhibited negative correlations with their counterparts in CSF, and the strongest correlations between CSF and plasma were observed for C-terminal NfL530 peptide and Coil 1 peptides NfL92 (NfL peptide AQLQDLNDR spanning residues 92–100), NfL101 and NfL117. These data together with NfL profiles in CSF and plasma indicate further studies are needed to understand the dynamics of NfL processing and its release from the cell to the blood.

#### CSF neurofilament light chain correlates with immunoassay measures

Neurofilament light chain IP–MS measures in CSF samples were compared to matched samples that were previously measured by Simoa Quanterix NfL immunoassay. We found that CSF IP–MS measures correlated well with immunoassay measures, with NfL324 peptide showing strongest correlation (Supplementary Fig. 2).

#### Plasma neurofilament light chain peptide measures correlate with age and body mass index

**Figure 3:**
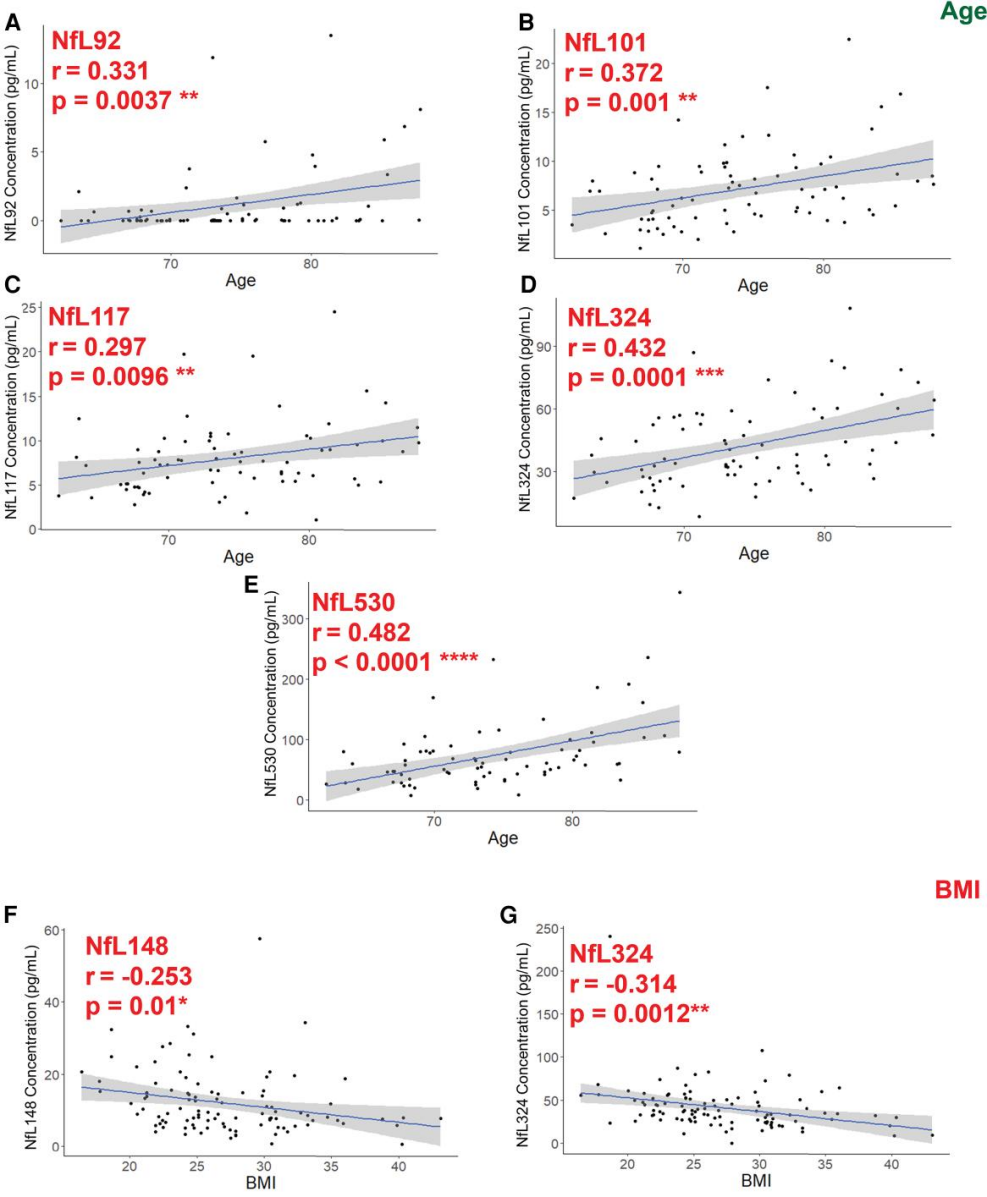
**IP–MS NfL plasma peptide correlations with age and BMI, two common confounding factors in blood plasma biomarker measures.** (**A–E**) Plasma NfL peptide concentrations are plotted against age, with NfL530 being most correlated (*r* = 0.48, *P* < 0.0001). (**F and G**) Plasma NfL peptide concentrations are plotted against BMI, with NfL324 most correlated with BMI (*r* = −0.314, *P* = 0.0012). Pearson correlation analysis was used to compare IP–MS measures with age and BMI, and statistical significance was determined by α = 0.05.

We observed statistically significant positive correlations for five of eight plasma NfL peptides (NfL92, NfL101, NfL117, NfL324 and NfL530, Fig. 3A–E) with age. Additionally, we observed statistically significant negative correlations for two of eight NfL peptides with BMI (NfL148 and NfL324), which agree with previously described relationships of NfL and BMI.

#### Clinical Dementia Rating–Sum of Boxes is associated with Tail subdomain peptide in CSF

We examined the relationship of CDR–SB scores with NfL IP–MS measures in plasma and CSF to determine the relationship of specific peptide measures to clinical measures of dementia. For CDR–SB > 0, C-terminal NfL530 peptide showed significant correlation in CSF, but not plasma. Coil 2 peptide NfL324 did not correlate in either plasma or CSF.

## Discussion

Neurofilament light chain proteoforms are promising candidates for neurodegeneration-specific biomarkers and has shown utility as a marker of neuronal damage across several pathologies. Immunoprecipitation–MS assays developed for other such fluid-based biomarkers (Aβ42/40, tau, phospho-tau, tau-MTBR) have shown advantages of high analytical precision, improved sensitivity and specificity of determining disease state and distinguishing tauopathies.^22-27^ Here, we present a novel method for IP and quantitation of NfL in blood by MS.

We show NfL has different profiles in CSF and blood plasma in matched samples, and statistically significantly increased peptides by amyloid group are different in CSF (Coil 1 peptides NfL92–165, C-terminal peptide NfL530) than in plasma (Coil 2B domain NfL324). To our knowledge this is the first MS assay to profile and quantify multiple NfL peptides in blood plasma. We replicated previous findings with respect to correlation of our plasma IP–MS NfL multi-peptide measures and previously confirmed covariates of plasma NfL including age (positively correlated, Fig. 3A–E) and BMI (negatively correlated, Fig. 3F and G). We hypothesized that there could be differences between amyloid-positive and amyloid-negative NfL profiles that could be elucidated by our assay. We did not discover disease-specific profiles for unmodified NfL peptides in plasma or CSF. Peptide profiles are similar for amyloid-positive and amyloid-negative individuals in CSF and plasma. Interestingly, we observed different peptide profiles when comparing averaged CSF and plasma NfL profiles (Fig. 2), where we observed overlapping Coil 1 peptides in plasma, and a higher ratio of C-terminal (NfL530) to Coil 2B (NfL324). Differences in NfL peptides were also observed in CSF-to-plasma ratios (Fig. 2C), which show higher relative levels of Coil 1 peptides in amyloid-positive CSF, and lower CSF-to-plasma ratio of C-terminal NfL530 relative to other peptides. These data could imply (i) a significant contribution from peripheral nervous system–derived NfL to the pool of centrally derived plasma NfL or (ii) increased truncation of NfL proteoforms or other post-translational modification (PTM) prior to release and dilution into the plasma. An additional possibility could be that plasma-derived NfL represents a pool that is isolated from extracellular vesicles, whereas NfL purified from CSF is a direct product of cell death or release of neurofilament. Extracellular vesicle–derived NfL has recently received increased interest and has been shown to be elevated in the first year of traumatic brain injury.^28^ The vesicular processing and export of NfL could result in different cleavage products than that of direct release from damaged cells.

**Figure 4:**
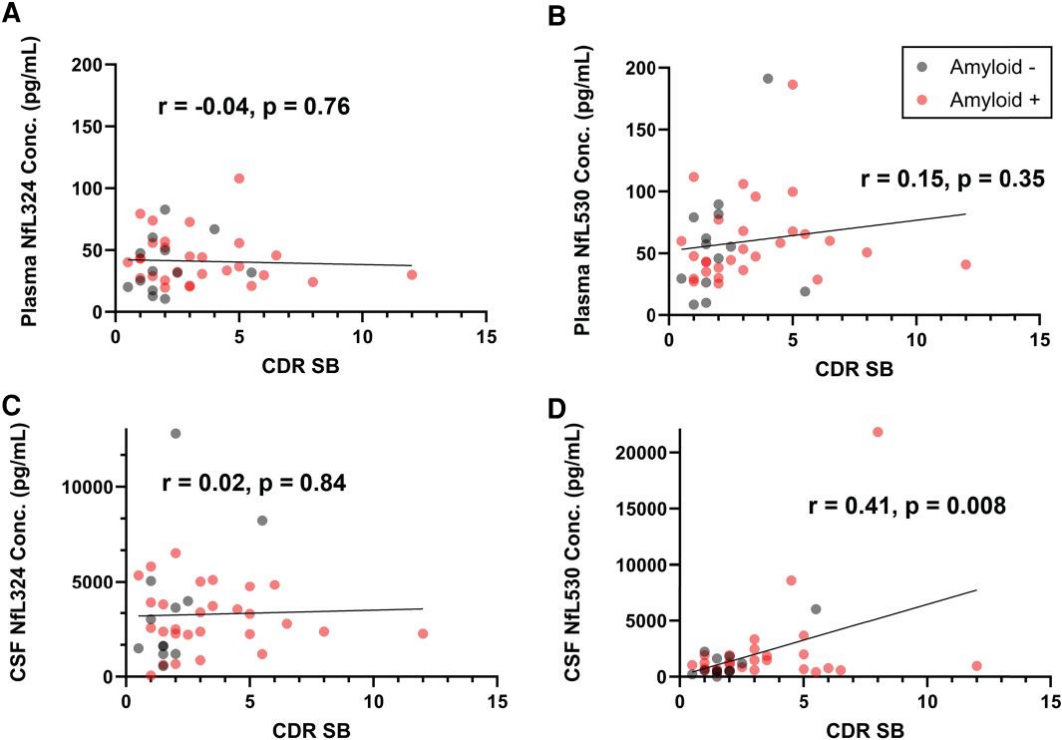
**Plasma and CSF NfL324 and NfL530 peptide concentration correlations with CDR–SB > 0.** NfL peptide concentrations are plotted against CDR–SB scores and coded by amyloid status given by Aβ42/40 (dark points are amyloid negative). Plasma and CSF NfL324 does not correlate with CDR–SB > 0 (**A** and **C**, respectively), whereas plasma NfL530 (**B**) shows modest correlation (*r* = 0.15) and CSF NfL530 (**D**) correlates with CDR–SB > 0 (*r* = 0.41, *P* = 0.008). Pearson correlations were used to compare CSF and plasma measures of NfL324 and NfL530, and statistical significance was determined by α = 0.05.

We sought to describe the relationship of plasma and CSF NfL to CDR–SB for potential benefit of increased gradation in clinical measures for samples analysed largely from individuals without clinical dementia. NfL324, which correlates best with immunoassay results (Supplementary Fig. 2),^9^ shows no correlation with CDR–SB > 0 in CSF or plasma (Fig. 4A and C), whereas the C-terminal NfL530 peptide appears to relate to CDR–SB > 0 in the CSF and shows modest correlation in plasma (Fig. 4B and D). This could imply differences in how NfL is processed, potential biological roles of the disordered C-terminal tail of NfL or point towards NfL species that could hold signatures more specific to dementia-involved neurodegeneration. It has been suggested previously that the C-terminal portion of NfL may be more susceptible to inclusion or aggregation upon inducing oxidative stress in cultured neurons.^29^

This study informs about physiologic processes and pathophysiological changes that occur in NfL prior to onset of advanced dementia due to Alzheimer’s disease in both CSF and plasma. This study has limitations in its relatively small size and lack of advanced clinical Alzheimer’s disease dementia and could be strengthened by examining larger cohort containing more examples of clinical Alzheimer’s disease. This was a retrospective characterization of neurofilament light chain in a cross-sectional study of amyloid kinetics in older participants and is an exploratory study of NfL proteoforms. We controlled for potential bias by maintaining blinding of researchers from the sample selection to data analysis stage. Additionally, the possibility of different NfL profiles between CSF and plasma should be tested in different neurodegenerative diseases and larger cohorts. These findings may aid in identifying more disease or neurodegeneration-specific isoforms for future biomarker use and offer the possibility of improved staging of Alzheimer’s disease and discrimination of neurodegenerative dementias.

## Supplementary Material

fcae247_Supplementary_Data

## Data Availability

Raw MS data and any data used for generating figures will be made available upon request by the corresponding author. In-house anti-NfL monoclonal antibodies will be made available upon reasonable request.
