# Supplementary material for: Multi-peptide characterization of plasma neurofilament light chain in preclinical and mild Alzheimer’s disease
Source: Brain Commun. 2024 Aug 20;6(4):fcae247. doi: 10.1093/braincomms/fcae247 (PMC11334934; doi:10.1093/braincomms/fcae247)
Supplement: fcae247_Supplementary_Data [file fcae247_supplementary_data.docx]

**Supplementary Material**


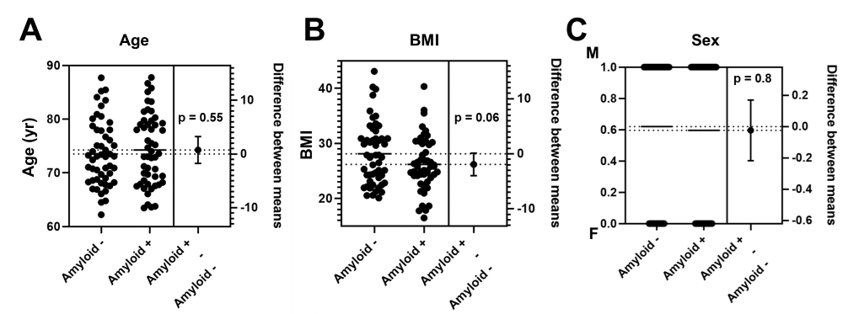


**Supplementary Figure 1** Cohort covariate analysis by amyloid status. Mean difference plots for (A) age, (B) BMI, and (C) sex comparisons for amyloid positive and negative participants show no statistically significant differences between amyloid groups by age (p=0.55), BMI (p=0.06), or sex (p=0.8); BMI is trending towards significantly lower in the amyloid + group. Statistical significance determined by unpaired t-test with Welch’s correction (α = 0.05).

**Supplementary Figure 2. IP-MS NfL CSF measure correlations with Quanterix immunoassay. (A)** Correlogram of CSF NfL peptide measures by IP-MS with immunoassay. **(B)** Scatterplots of CSF NfL IP-MS peptide measures against matched measures of CSF NfL via Quanterix immunoassay indicate NfL324 has the highest correlation (r = 0.77) and NfL284 the lowest correlation (r = 0.19) compared to the Uman/Simoa immunoassay. Pearson correlations were used to compare immunoassay and CSF IP-MS measures of NfL, and correlations are provided in bold within each subpanel of **(B)**.


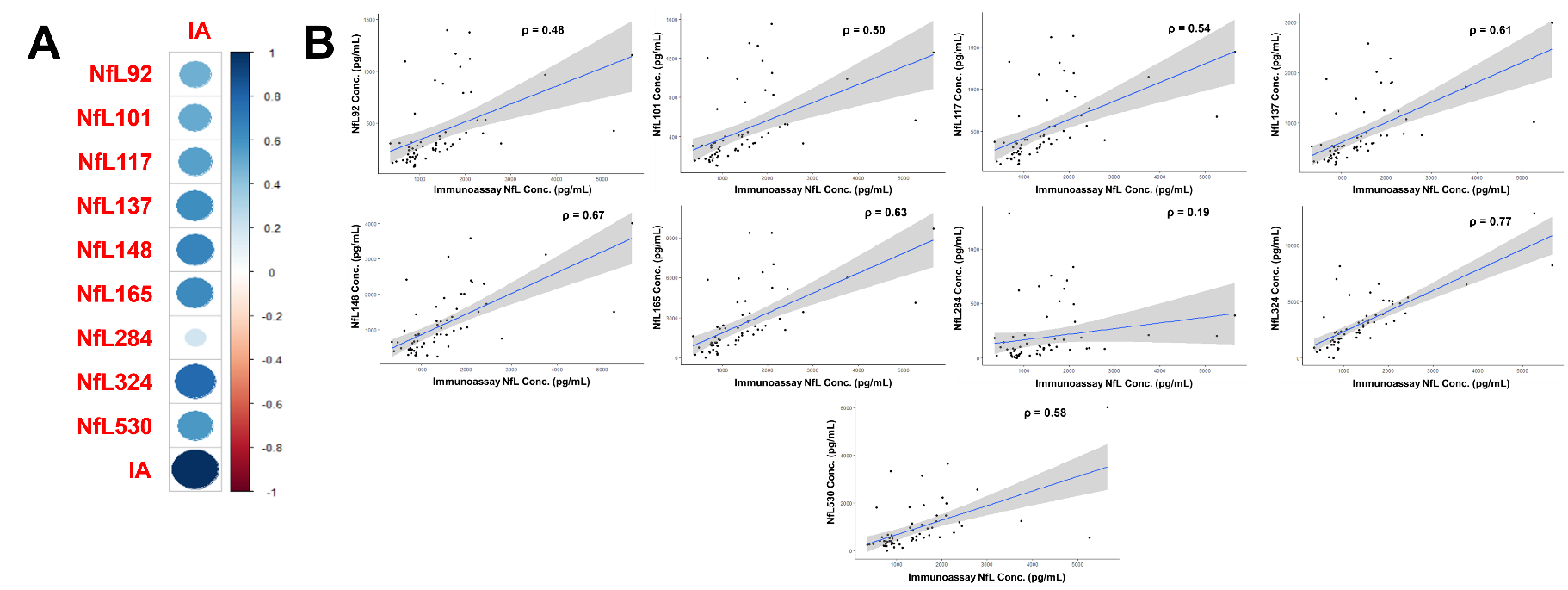


| Characteristic |  | Amyloid - (n=50) | Amyloid + (n=52) |  | All (n=102) |
| --- | --- | --- | --- | --- | --- |
|  |  |  |  |  |  |
| Age (years ± SD, range) |  | 73.5 ± 5.9, 25.5 | 74.3 ± 6.6, 24.3 |  | 73.9 ± 6.3, 25.6 |
| Sex (n, %F) |  | 19, 38% | 21, 40% |  | 40, 39% |
| BMI (avg ± SD, range) |  | 28.1 ± 5.6, 23.01 | 26.2 ± 4.7, 23.9 |  | 27.2 ± 5.3, 26.6 |
| ApoE 4 (n, %) |  | 4, 8% | 27, 52% |  | 31, 30% |
| CDR(0/0.5/1, %>0) |  | 34/11/1, 24% | 23/18/7, 48% |  | 57/29/8, 36% |
| CDR Sum of Boxes (avg ± SD) |  | 0.5 ± 1.1 | 1.8 ± 2.5 |  | 1.1 ± 2.0 |
| MMSE (avg ± SD) |  | 28.6 ± 1.8 | 26.9 ± 3.2 |  | 27.7 ± 2.5 |

**Supplementary Table 1.** Validation cohort demographics. Abbreviations: %F denotes percent of female participants, BMI denotes body mass index, CDR denotes clinical dementia rating, MMSE denotes Mini-Mental State Examination, SD denotes standard deviation.
